# Supplementary material for: Nursing supervisors’ perspectives on student preparedness before clinical placements- a focus group study
Source: PLoS One. 2021 May 28;16(5):e0252483. doi: 10.1371/journal.pone.0252483 (PMC8162812; doi:10.1371/journal.pone.0252483)
Supplement: S1 File — (DOCX) [file pone.0252483.s001.docx]

| **Theme 1. Practical skills** |
| --- |
| What do you think when I say «practical skills»? In relation to registered nurse students? In relation to intellectual disability nurse students?  Which practical skills are most important? Which practical skills are needed in your ward?  What are your expectations regarding students’ practical skills prior to clinical placement in your ward? |
| **Theme 2. Procedures and preparedness** |
| How would you describe a well-prepared student?  Are there specific procedures students should be better prepared for?  Which procedures do you think students should learn in the university college? In clinical placement? |
| **Theme 3. Supervision** |
| Could you please discuss your experiences related to clinical supervision.  Please describe a concrete situation where you expected a level of competence in the student, where this was not the case.  Please describe a concrete situation where the students’ level of competence was higher than expected. |
