# Supplementary material for: Nursing supervisors’ perspectives on student preparedness before clinical placements- a focus group study
Source: PLoS One. 2021 May 28;16(5):e0252483. doi: 10.1371/journal.pone.0252483 (PMC8162812; doi:10.1371/journal.pone.0252483)
Supplement: Attachment — Submitted filename: PONE-D-21-03784_reviewer.pdf [file pone.0252483.s002.pdf]

PLOS ONE

Nursing supervisors’ perspectives on student preparedness before clinical placements- a focus group study

--Manuscript Draft--

**Manuscript Number:**	PONE-D-21-03784
**Article Type:**	Research Article
**Full Title:**	Nursing supervisors’ perspectives on student preparedness before clinical placements- a focus group study
**Short Title:**	Student preparedness for clinical practice
**Corresponding Author:**	Ann-Chatrin L Leonardsen<br>Østfold University College<br>Fredrikstad, Kråkerøy NORWAY
**Keywords:**	nursing student, clinical placement, supervision, expectations, preparedness, focus group
**Abstract:**	Clinical placements and supervisions is an integral part of nursing education internationally. There are significant differences between students’ expectations of clinical learning and their fulfillment. Few studies have focused on supervisors’ perspectives on clinical placements. The objective of this study was to explore supervisors’ perspectives on student preparedness for clinical placements. Methods: The study was conducted in a county in Southeastern-Norway, with 317.000 inhabitants, and within one hospital and one university college catchment area. Focus group interviews were conducted in the periode August to December 2018. Data were analyzed using Hsieh and Shannon’s conventional content analysis. Results: 34 nursing supervisors participated, three intellectual disability nurses and 31 registered nurses, working in four different primary healthcare wards and four different hospital wards. Participants age ranged from 23 to 58 years, one male only. Through the analysis we derived the category ‘Shared responsibility for preparation’ with subcategories a) Individual initiative, and b) University college facilitation. Conclusions: Findings indicate that there is a gap between supervisors’ expectations and reality regarding students’ preparedness for clinical placements. Moreover, supervisors did not seem to focus on their own role in student preparedness.
**Order of Authors:**	Ann-Chatrin L Leonardsen
	Siri E Brynhildsen
	Mette E Hansen
	Vigdis Abrahamsen A. Grondahl
**Additional Information:**	
**Question**	**Response**
**Financial Disclosure**<br><br>Enter a financial disclosure statement that describes the sources of funding for the work included in this submission. Review the submission guidelines for detailed requirements. View published research articles from *PLOS ONE* for specific examples.<br><br>This statement is required for submission and **will appear in the published article** if the submission is accepted. Please make	ACL received fundings from Østfold University College (https://www.hiof.no/).<br>The funders had no role in study design, data collection and analysis, decision to publish, or preparation of the manuscript.

Powered by Editorial Manager® and ProduXion Manager® from Aries Systems Corporation

sure it is accurate.<br><br>**Unfunded studies**<br>Enter: *The author(s) received no specific funding for this work.*<br><br>**Funded studies**<br>Enter a statement with the following details:<br>• Initials of the authors who received each award<br>• Grant numbers awarded to each author<br>• The full name of each funder<br>• URL of each funder website<br>• Did the sponsors or funders play any role in the study design, data collection and analysis, decision to publish, or preparation of the manuscript?<br>• **NO** - Include this sentence at the end of your statement: *The funders had no role in study design, data collection and analysis, decision to publish, or preparation of the manuscript.*<br>• **YES** - Specify the role(s) played.<br><br>* typeset	
**Competing Interests**<br><br>Use the instructions below to enter a competing interest statement for this submission. On behalf of all authors, disclose any competing interests that could be perceived to bias this work—acknowledging all financial support and any other relevant financial or non-financial competing interests.<br><br>This statement is required for submission and **will appear in the published article** if the submission is accepted. Please make sure it is accurate and that any funding sources listed in your Funding Information later in the submission form are also declared in your Financial Disclosure statement.<br><br>View published research articles from *PLOS ONE* for specific examples.	The authors have declared that no competing interests exist.

Powered by Editorial Manager® and ProduXion Manager® from Aries Systems Corporation

**NO authors have competing interests**<br><br>Enter: *The authors have declared that no competing interests exist.*<br><br>**Authors with competing interests**<br><br>Enter competing interest details beginning with this statement:<br><br>*I have read the journal's policy and the authors of this manuscript have the following competing interests: [insert competing interests here]*<br><br>* typeset	
**Ethics Statement**<br><br>Enter an ethics statement for this submission. This statement is required if the study involved:<br><br>• Human participants<br>• Human specimens or tissue<br>• Vertebrate animals or cephalopods<br>• Vertebrate embryos or tissues<br>• Field research<br><br>Write "N/A" if the submission does not require an ethics statement.<br><br>General guidance is provided below. Consult the submission guidelines for detailed instructions. **Make sure that all information entered here is included in the Methods section of the manuscript.**	The study was approved by the Norwegian Center for Research Data (NSD) (Ref. no.951914)

**Format for specific study types**<br><br>**Human Subject Research (involving human participants and/or tissue)**<br>• Give the name of the institutional review board or ethics committee that approved the study<br>• Include the approval number and/or a statement indicating approval of this research<br>• Indicate the form of consent obtained (written/oral) or the reason that consent was not obtained (e.g. the data were analyzed anonymously)<br><br>**Animal Research (involving vertebrate animals, embryos or tissues)**<br>• Provide the name of the Institutional Animal Care and Use Committee (IACUC) or other relevant ethics board that reviewed the study protocol, and indicate whether they approved this research or granted a formal waiver of ethical approval<br>• Include an approval number if one was obtained<br>• If the study involved *non-human primates*, add *additional details* about animal welfare and steps taken to ameliorate suffering<br>• If anesthesia, euthanasia, or any kind of animal sacrifice is part of the study, include briefly which substances and/or methods were applied<br><br>**Field Research**<br><br>Include the following details if this study involves the collection of plant, animal, or other materials from a natural setting:<br>• Field permit number<br>• Name of the institution or relevant body that granted permission	
**Data Availability**<br><br>Authors are required to make all data underlying the findings described fully available, without restriction, and from the time of publication. PLOS allows rare exceptions to address legal and ethical concerns. See the PLOS Data Policy and FAQ for detailed information.	No - some restrictions will apply

Powered by Editorial Manager® and ProduXion Manager® from Aries Systems Corporation

A Data Availability Statement describing where the data can be found is required at submission. Your answers to this question constitute the Data Availability Statement and **will be published in the article**, if accepted.<br><br>**Important:** Stating 'data available on request from the author' is not sufficient. If your data are only available upon request, select 'No' for the first question and explain your exceptional situation in the text box.<br><br>Do the authors confirm that all data underlying the findings described in their manuscript are fully available without restriction?	
**Describe where the data may be found in full sentences. If you are copying our sample text, replace any instances of XXX with the appropriate details.**<br><br>• If the data are **held or will be held in a public repository**, include URLs, accession numbers or DOIs. If this information will only be available after acceptance, indicate this by ticking the box below. For example: *All XXX files are available from the XXX database (accession number(s) XXX, XXX.*).<br>• If the data are all contained **within the manuscript and/or Supporting Information files**, enter the following: *All relevant data are within the manuscript and its Supporting Information files.*<br>• If neither of these applies but you are able to provide **details of access elsewhere**, with or without limitations, please do so. For example:<br><br>*Data cannot be shared publicly because of [XXX]. Data are available from the XXX Institutional Data Access / Ethics Committee (contact via XXX) for researchers who meet the criteria for access to confidential data.*<br><br>*The data underlying the results presented in the study are available from (include the name of the third party*	Data cannot be shared publicly due to confidentiality issues, and the possibility that individuals may be recognized in the data. Data may be shared upon reasonable request to the first author.

Powered by Editorial Manager® and ProduXion Manager® from Aries Systems Corporation

and contact information or URL).<br>• This text is appropriate if the data are owned by a third party and authors do not have permission to share the data.<br><br>* typeset	
Additional data availability information:	Tick here if your circumstances are not covered by the questions above and you need the journal's help to make your data available.

Manuscript Click here to access/download;Manuscript;Manuscript.docx

Title: **Nursing supervisors' perspectives on student preparedness before clinical placements- a focus group study**

Ann-Chatrin Leonardsen[1,2*], Siri Brynhildsen[1], Mette T. Hansen[1], Vigdis A. Grøndahl[1]

1. Østfold University College, Postal box code 700, 1757 Halden, Norway
2. Østfold Hospital Trust, Postal box code 300, 1714 Grålum, Norway

Corresponding and first author: E-mail: ann.c.leonardsen(at)hiof.no

Abstract

Clinical placements and supervisions is an integral part of nursing education internationally. There are significant differences between students' expectations of clinical learning and their fulfillment. Few studies have focused on supervisors' perspectives on clinical placements. The objective of this study was to explore supervisors' perspectives on student preparedness for clinical placements. **Methods:** The study was conducted in a county in Southeastern-Norway, with 317.000 inhabitants, and within one hospital and one university college catchment area. Focus group interviews were conducted in the periode August to December 2018. Data were analyzed using Hsieh and Shannon's conventional content analysis. **Results:** 34 nursing supervisors participated, three intellectual disability nurses and 31 registered nurses, working in four different primary healthcare wards and four different hospital wards. Participants age ranged from 23 to 58 years, one male only. Through the analysis we derived the category 'Shared responsibility for preparation' with subcategories a) Individual initiative, and b) University college facilitation. **Conclusions:** Findings indicate that there is a gap between supervisors' expectations and reality regarding students' preparedness for clinical placements. Moreover, supervisors did not seem to focus on their own role in student preparedness.

INTRODUCTION

Nurse education in Europe is characterized by different structures, standards and approaches to the relationship between theoretical and practice based learning (1, 2). The European Commission has, through the Bologna Treaty Process, promoted greater harmonisation of

educational systems in the European area (3). Such harmonisation seeks to increase the employment and educational mobility of nursing staff and students.

Nursing (RN) and intellectual disability nursing (IDN) students need to employ their knowledge and skills in clinical environments to acquire the required qualifications for taking care of patients, and their success depends to a great extent on efficient clinical training (4, 5). Clinical supervision is widely used as a formal process of professional support for undergraduate nursing students (6), and assists the development of professional competence and confidence in nursing students, ultimately enhancing the provision of quality patient care (7). The support provided through clinical supervision helps the students to link theoretical knowledge provided through lectures, with patient care in the clinical environment (6, 8, 9). Clinical supervision is beneficial to the students by directly observing students in the clinical facility, building a supportive relationship between the supervisor and the student (10). The benefit of this supportive relationship is that it optimizes teaching and learning (11). Lack of this supportive supervisor–supervisee relationship has been found to result in negative clinical learning experiences such as poor communication (12). Scholars allude to strengthening of clinical supervision in order to contribute to the student's personal growth, for example good communication skills (8, 11).

Expectations may be understood as reasonable likelihood that something will occur (13). Expectations are linked with motivational (interests) and cognitive (assessment) aspects of behavior, and they carry important repercussions in the processes of decision-making, persistence in certain behaviors, and academic and workplace performance, among others (14-16). Statistically significant differences have for example been found between the initial expectations and their fulfillment at the end of the academic year for all the factors and in all years of the bachelor degree program (17).

University programs expect students to be actively engaged in contributing to their experience and determining appropriate learning outcomes. In the clinical context, this requires that educators collaborate with students' to identify their learning needs, ensure they are provided with opportunities to be self-directed and develop a learning experience that supports the students' level of interest. For the student, this requires that they are well prepared in terms of foundational knowledge and attitudes at the commencement of practice education. This infers that they accept a share of the responsibility for planning and preparing for the learning experience (18).

Research on supervisors perspectives, as the ones who direct the students to gain professionally related skills in the clinical environment, is limited (18, 19). Consequently, the aim of this study was to explore nursing- and intellectual disability nursing supervisors' perspectives on students' preparedness to clinical placements.

Materials and methods

Design

The study is in-line with the Consolidated criteria for reporting qualitative research – COREQ (20). The study had a qualitative design, utilizing focus group interviews to explore supervisors' perspectives on students' preparedness for clinical placements. A qualitative approach is appropriate when aiming to explore how individuals experience a phenomenon, dependent on their background, interests and interpretation (21). Focus groups are appropriate when exploring experiences and perceptions, enabling further development of these through group discussions among participants. During focus group interviews interaction, dialogue, and shared reflections between the participants, can increase and deepen the understanding of the phenomenon that is explored (22, 23).

Setting

The study was conducted in a county in south-eastern Norway concisting of 18 municipalities, and within the cathcment area of one hospital trust that covers 317 000 inhabitants. Within the municipalities there are five decentralized acute care wards and five casualties. Moreover, there are 34 nursing homes, and more than 4o home based nursing wards, in addition to institutional services for persons with intellectual and physical disabilities. The county adhere to one university college, which provides bachelor degree education for nursing- and intellectual disability nursing students. A total of approximately 1700 clincial placements are carried out every year, and approximately 160 registered nurses (RN) and between 45 and 95 intellectual disability nurses (IDN) finish their bachelor's degree from the university college each year.

Sample

We chose a purposefull sampling strategy where supervisors from all the different types of clinical placement wards were invited to participate. We aimed at including supervisors from both primary healthcare services and different hospital wards. Pragmatically, we aimed at including participants to eight focus group interviews. We randomly selected two medical and two surgical hospital wards respectively, as well as four wards in primary healthcare services. Managers in each ward selected participants assumed to be information rich, experienced with supervision of RN/IDN students. Inclusion criteria were; RN/IDNs who had supervised bachelor students within the last two years.

The supervisors were given verbal and written information about the study and asked to participate by their manager. The supervisors who wanted to participate, signed the informed consent.

Interview guide

A semi structured interviewguide was developed based on the respective bachelor program's curriculum, informal feedback from teachers at the university college and from supervisors, also including the researchers' own experiences and competencies from education and the clinical field. The interview guide was piloted in the research group. The questions were used to guide the focus group interviews, and started with items concerning biographical data. The interview guide continued with open questions (e.g. *'Could you please describe practical skills and your experiences of how the students learn these skills?'*). In order to obtain rich and meaningful data, probing questions were sometimes asked by the researchers to extend or narrow the field of interest (e.g. *'Could you please elaborate on that?'*, and *'What about the rest of you? How are your experiences?'*).

Procedure

The focus group interviews were conducted nearby the participants' workplace in an office or meeting-room away from the actual wards. The interviews started with an open dialogue where the participants were encouraged to freely discuss their experiences related to supervision of nursing/ID nursing students during clinical placement. The interview guide was then used as a «checklist» to the ensure that the themes were covered. The focus group interviews were conducted by two and two participants of the research group, consisting of RN and IDN educators and clinicians, some with a PhD and some without (one male). Their roles were as active interviewer and observer respectively. All researchers were experienced RN/IDNs, with several years of professional practice, and/or several years of experience as educators.

The focus group interviews were conducted in the periode August to December 2018. The interviews lasted from 30 to 70 minutes (mean 50 minutes). The interviews were digitally recorded, and transcribed verbatim by an external transcriber, who had signed a non disclosure agreement. The recordings were deleted after transcription.

Analysis

Data was analysed according to Hsieh and Shannon's conventional content analysis, which is appropriate when the aim of the study is to describe an under- or unexplored phenomenon (24). The analysis started with reading the transcripts repeatedly to obtain a sense of the whole (all authors). Then the transcripts were read word by word, and exact words that appeared to capture key thoughts or concepts were highlighted (two of the authors). Notes were made to catch the first impressions, thoughts and initial analysis, and subsequently codes were derived based on more than one key thought (three of the authors). During the analysis, the transcripts were included in a table. Key words were marked yellow. Labels for codes were then transferred to the next column (initial coding scheme), and categories placed in the next column. All authors (all authors) read the transcripts, and the preliminary derived codes were discussed untill consensus was reached. The codes were then sorted into different categories depending on how they were related and linked (three of the authors). The development of categories were also discussed among all authors untill consensus was reached. This was an iterative process, moving back and forth from transcripts to codes to categories.

Ethical consideration

The study was approved by the Norwegian Center for Research Data (NSD) (Ref. no.951914), and was conducted in-line with recommendations in the Declaration of Helsinki (25). The supervisors received oral and written information about the study purpose, and delivered signed written consents to participate. Due to the nature of a focus group, it was not possible to withdraw from the study. Participation was voluntary.

Results

A total of 34 RN/IDN student supervisors participated, 31 RNs and three IDNs respectively. The supervisors worked in psyciatric and substance abuse care, in home healthcare, in housing for disabled people, in a nursing home in primary healthcare, and in medical and surgical wards, including the children's ward in hospital. The supervisors were from 23 to 58 years of age, mean age 39.5 years. They had worked as RN/IDN from one to 35 years, and at the current workplace between six weeks and 20 years. Sixteen of the 34 supervisors had completed a formal supervisor course. More than two third (25 out of 34) of the supervisors had been responsible for many students during the years. Only one of the supervisors was a man.

Through the analysis we derived the category 'Shared responsibility for preparation' with subcategories a) Individual initiative, and b) University college facilitation. Illustrative quotes are marked with focus group (FG) number.

Shared responsibility for preparation

All of the focus group participants found preparedness as essential for students to get the most out of their clinical placement, both personally and related to learning outcomes. For students to be prepared for clinical placement supervisors assumed a shared responsibility between the student and the university college. They expected the student to be acquainted with the patient groups as well as possible learning outcomes for the specific clinical placement. Moreover, they expected that the university college provided the students with theoretical introduction and practical exercises in different practical procedures. These aspects were discussed and emphasized as important in all focus groups, across RN/IDN specialization and primary- and hospital healthcare wards. In addition, there were no disagreements between participants within the focus groups regarding these aspects.

Individual initiative

Throughout, there was an agreement in all focus groups that the student had an individual responsibility to be prepared, and hereby needed individual initiative. The supervisors in all focus groups emphasized that the students should reflect on their role as a student. Moreover, supervisors expected students to have insight into the learning outcomes related to the specific educational semester, as well as the potential learning opportunities at the specific ward. Most focus groups (FG) also expressed increasing expectations of the students' preparedness from first to the last semester of the education program. One of the supervisors in FG 4 stated;

«That he thinks through what is expected as a student. That he knows what he is good at, and that he is prepared for those things is important for the third year.» (FG 4)

This was supported by the other supervisors in the group. Supervisors in all groups also found it important that students had knowledge about different relevant diseases in the actual placement, both regarding pathology, medical treatment and nursing care needed in the specific group of patients. As stated in FG 6:

«That they are prepared and have knowledge about the patient group they will meet.» (FG 6)

Some of the wards had developed an information letter about the placement that students got access to before the placement periode. Here, supervisors expected the students to have read the information, even though this was not always the case. The supervisors in many of the focus groups also would like the students to visit the ward in advance.

In some of the focus groups, supervisors expressed that it was an advantage when the students had work experience from health or social services before starting their education and also that they worked during their studies. This was particularly emphasized as an advantage prior to clinical placements in substance abuse care and mental health work;

«To have som psychiatric experience or know a little of relationships, man, wholeness.» (FG 9)

University college facilitation

In all focus groups, supervisors expressed that they assumed that the university college is responsible for facilitating for students to access both theoretical and practical knowledge. They all expected that the students had been introduced to, and given the opportunity to perform different practical skills and procedures at least once at the university college. Hygienic principles and aseptic procedures were examplified as knowledge that could be transferred to many procedures, and the supervisors assumed that the university college enabled students to do so. Moreover, they pointed out that by letting the students practice on each other, one might perhaps lessen the discomfort one can feel when the procedures are to be performed on a patient for the first time. In FG 3 this was prompted:

«*That they experience what it is like to have your teeth brushed by someone else, or be fed or having a bed bath.» (FG 3)*

and further

«Then the training can be done during the clinical placement, you get to try it several times, like injections, or, yes, wound care» (FG 3)

The supervisors also expected that the university prepared the students for various clinical placements. For example, before clinical placement in surgical wards the participants expected the students to have knowledge of preoperative and postoperative nursing. A supervisor in FG 10 said:

«… then I expect that they have had lectures in pre and postoperative nursing. They should know what to look for when someone has just had surgery. What is the postoperative nursing, elimination, pain…..» (FG 10)

Prior to clinical placement in mental health care, the supervisors expected the students to have knowledge of e.g. guardianship, law of coercion and force, and also of the most common drugs used in mental health treatment. FG 7 agreed on this statement:

«What is psychiatry? It is no longer just the medication. If they do not want to take their medicines, we can not force them, and then we have to do something else, and what do we do then?» (FG 7)

In several of the focus groups, the supervisors claimed that the university college had to prepare the students to be creative and adapt the nursing care to the current situation in the different care settings. This was espescially emphasized when the students were in clinical placements in the patient`s home in home health care services. In FG 4 this was concretized:

«Because we can enter a home and there is a man with a cardiac arrest in bed, what do you do?» (FG 4)

Discussion

Our findings show that supervisors had expectations regarding students' individual initiative to be prepared for clinical placement, as well as the university college facilitation of such preparedness. The focus groups consisted of participants from various primary healthcare wards, as well as hospital wards, and both RNs and IDNs, still there were no disagreements on these issues.

Supervisors perceived that students should take their part of the responsibility for being prepared for clinical placements. Moreover, they emhasized that this would require individual initiative, since the students would have to reflect, search for relevant literature regarding specific conditions or even visit the actual ward prior to the placement periode. This is in-line with findings from e.g. Chipchase et al (18), who emhasized that students should accept a share of the responsibility for planning and preparing for the learning experience. Webb et al. (26) also found that supervisors call for students who are well prepared theoretically in front of clinical placements. In a recent study, supervisors rated professionalism and willingness as the most important characteristics in nursing students, followed by personal attributes. The authors concluded that further strengthening learning opportunities related to these characteristics in the curriculum may improve the students' preparedness in clinical learning (27).

The supervisors also discussed the need to combine theoretical knowledge and practical skills, to be able to meet patients as whole human beings. According to Bloom (28) there are three domains of learning: 1. Knowledge (cognitive, mental skills), 2. Skills (psychomotor, manual or physical skills), and 3. Attitude (affective, growth in feelings). The clinical environment is a place for students to practice and gain both confidence and competence in all three of these domains (29). Clinical training is regarded as an integral part of learning and education in nursing(5, 30). Furthermore, the clinical learning environment plays an important role in turning nursing students into professionals and preparing them to function as nurses (31). Nevertheless, supervisors expected that students had both knowledge and skills *before* their clinical placement.

The supervisors expected the university college to provide students with both theoretical knowledge and practical training relevant for the specific clinical placement. In a recent study across primary and tertiary healthcare in the same county, we found that

RNs/IDNs perceived that students should learn technical skills in the university college first, and then get more training in clinical placement (32). This was supported by findings from the focus group discussions in the current study.

Earlier studies have emphasized that supervising nursing students affects RNs' daily work to different degrees, depending on students' knowledge, progress and willingness to learn, but also on the supervisors themselves (33, 34). Moreover, studies indicate that the nursing students are not psychologically prepared for clinical placements (35, 36). In the current study, issues related to the supervisors themselves were not discussed. The gap between supervisors' expectations, and students' preparedness could lead to challenges both regarding the establishment of a constructive supervisor-supervisee relationship, the supervisors' experience of their role as a burden, and to the students' clinical learning. According to Kalyani et al. (37), educational policymakers should focus on increasing supervisors' influence on management, education and clinical education, as well as teaching positive and constructive strategies to cope with inadequate educational contexts. Donough et al. (38) also suggest a need for continuous professional development for clinical supervisors by means of in-service training.

Limitations

First, the data was collected by seven different interviewers., which may have introduced a researcher-bias. Still, they all had experience in conducting interviews, and had long experience from both clinical practice as RNs/IDNs and educators. In addition, to minimize the possible differences, the interview guide was discussed in advance among the researchers to reach a joint understanding of the questions and how to perform the focus group interviews.

Second, in this study, both RNs and IDNs were included, but few IDNs participated. IDNs are authorizeid healthcare personnel and work in different clinical settings. Inclusion of

more IDNs might have proved additional data, but even if they were few in numbers, they contributed with important experiences from being supervisors to IDN students.

Credibility was ensured through several and iterative analysis and discussions both between the authors and between research group members. The research group consists of 12 IDNs and RNs from both clinical wards and the university college. The supervisors represented both RNs and IDNs, from a variety of clinical wards both in primary- and hospital healthcare, indicating transferability of findings. To ensure dependability, the first author verified the authenticity of the recordings of the interviews against the transcripts.

Conclusion

Fidnings indictate the supervisors across primary and tertiary healthcare have expectations to students' individual initiative to get prepared for clinical placement, as well as the educational institutions' fascilitation thereof. In contrast, they did not discuss their own role in getting students prepared for clinical placements, or on how they could support students at the beginning of the placement periode. Our findings indicate that there is a gap between supervisors expectations and reality, which could impact both their relationship to the student, as well as the students' learning process. Clinical placement quality improvement initatives should focus on closing this gap.

Relevance to clinical practice

Further studies should focus on how supervisors could contribute to students' preparedness, together with the educational institutions. Moreover, students' and supervisors' perspectives should be compared and efforts made to align these, aiming for expectations to be in-line with reality.

Author's contribution

SB and VAG performed the initial analysis. SB, VAG and ACL discussed the initial analysis, and identified initial categories. All authors discussed these untill consensus was reached. ACLL wrote a first edition of the full manuscript, of which all of the co-authors (SB, MTH and VAG) participated in the drafting and critical revision of the manuscript for important intellectual content, as well as approval of the final submitted version of the manuscript. All co-authors (ACL, SB, MTH and VAG) contributed in the conception and planning of the work that led to the manuscript, analysis and interpretation of the data. All authors have read and approved the final version of the manuscript.

Acknowledgements

Inger Hjelmeland, Ina Kristin Blågestad, Anne Grethe Gregersen, Lars Gunheim-Hatland, Anne Herwander Kvarsnes, Mona Martinsen, Richard Olsen, and Wenche Charlotte Hansen are acknowledged for participating in the planning of the study, as well as in development of the interview guide. In addition, Anne Grethe Gregersen and Lars Gunheim-Hatland are acknowledged for participating in the focus group interviews.

Conflicts of interest

The authors declare no conflicts of interest.

Funding

The study was funded by collaborational fundings from Østfold University College.

References

1. Salminen L, Stolt M, Saarikoski M, Suikkala A, Vaartio H, Leino-Kilpi H. Future challenges for nursing eductaion- A European perspective. Nurse Educ Today. 2009;30(233-238).
2. Suhonen R, Saarikoski M, Leino-Kilp H. Cross-cultural research: a discussion paper. Int J Nurs Stud. 2009;45:593-602.

3. World Health Organization. European Union Standards for Nursing and Midwifery: Information for Accession Countries. 2009.Available from: https://www.euro.who.int/en/health-topics/Health-systems/nursing-and-midwifery/publications/2009/european-union-standards-for-nursing-and-midwifery-information-for-accession-countries.
4. Henderson A, Twentyman M, Heel A. Students' perception of the psycho-social clinical learning environment: an evaluation of placement models. Nurse Educ Today. 2006;26:564-71.
5. Dadgaran I, Parvizy S, Peyrovi H. Nursing students' views of sociocultural factors in clinical learning: a qualitative content analysis. Jpn J Nurs Sci. 2013;10:1-9.
6. Franklin N. Clinical supervision in undergraduate nursing students: a review of the literature. J Business Educ Scholarship Teach. 2013;7(1):34-42.
7. Muthathi A, Thurling C, Armstrong S. Through the eyes of the student: Best practices in clinical facilitation. Curationis. 2017;40(1):8.
8. Amsrud K, Lyberg L, Severinsson E. The influence of clinical supervision and its potential for enhancing patient safety- undergraduate nursing students views. J Nurs Educ Pract. 2015;5(6):87-95.
9. Brunero S, Stein-Parbury J. The effectiveness of clinical supervision in nursing: an evidence based literature review. Austr J Adv Nurs. 2011;25(3):86-94.
10. Bifarin O, Stonehouse D. Clinical supervision: an important part of every nurses' practice. Br J Nurs. 2017;26(6):331-5.
11. Nabolsia M, Zumotb A, Wardamc L, Abu-Moghli F. The experience of Jordanian nursing students in their clinical practice. Procedia- Soc Behav Sci. 2012;46(2012):5849-57.
12. Miller C. The work, preparation and development of nursing clinical teachers 2012. Available from: http://www.vuir.vu.edu.au/25824/1/Clive%20Leslie%20Miller.pdf.
13. Edberg A, Andersson P. The shift from a medical to a nursing orientation: a comparison of Swedish nursing students' expectations when entering the nursing degree programme in 2003 and 2013. Nurse Educ Today. 2015;35:78-83.
14. Cowen K, Hubbard L, Hancock D. Expectations and experiences of nursing students in clinical courses: a descriptive study. Nurse Educ Today. 2018;67:15-20.
15. Hamshire C, Willgoss T, Wibberley C. What are reasonable expectations? Healthcare students perceptions of their programmes in the North West of England. Nurse Educ Today. 2013;33(2):173-9.
16. Lovric R, Prlic N, Milutinovic D, Marjanac I, Zvanut B. Changes in nursing students' expectations of nursing clinical faculties competences: a longitudinal, mixed methods study. Nurse Educ Today. 2017;59:38-44.
17. Hidalgo-Blanco M, Puig-Llobet M, Lluch-Canu M. Expectations of nursing degree students: A longitudinal analysis. Nurs Educ Today. 2020.
18. Chipchase L, Buttrum P, Dunwoodie R, Hill AJ, Mandrusiak A, Moran M. Characteristics of student preparedness for clinical learning: clinical educator perspectives using the Delphi approach. VBMC Med Euc. 2012;12(1):112.
19. Lewallen L, De Brew J. Succesful and unsuccesful clinical nursing students. J Nurs Educ. 2012;51(7):389-95.
20. Tong A, Sainsbury P, Craig J. Consolidated criteria for reporting qualitative research (COREQ): a 32-item checklist for interviews and focus groups. Int J Qual Health Care. 2007;19|(6):349-57.
21. Langdridge D. Phenomenological psychology: theory, research and method. Harlow, UK: Pearson Education; 2007.
22. Lindseth A, Norberg A. A phenomenological hermeneutical method for researching lived experience. J Adv Nurs. 2004;18:145-53.
23. Beck D, Polit C. Nursing Research. 11th ed. Wolters Kluwer Health2018.
24. Hsieh H, Shannon S. Three approaches to qualitative content analysis. Qual Health Res. 2005;15(9):1277-88.
25. World Medical Association. Declaration of Helsinki- Ethical Principles for Medical Research Involving Human Subjects 2015. Available from: http://www.wma.net/en/30publications/10policies/b3.

26. Webb C, Shakespeare P. Judgements about mentoring relationships in nurse education. Nurse Education Today. 2008;28(5):563-671.
27. Banneheke H, Dnadarajah V, Ramamurthy S. Student preparedness characteristics important for clinical learning: perpsectives of supervisors from medicine, pharmacy and nursing. BMC Med Educ. 2017;17:130.
28. Anderson L, Karthwohl D, Bloom B. A taxonomy for learning, teaching and assessing: a revision of Bloom's taxonomy of educational objectives. Boston: Allyn& Bacon; 2001.
29. Delany C, Molloy E. Learning and teaching in clinical context: a practical guide. Chatswood: Elsevier; 2018.
30. Egan T, Jayae C. Communities of clinical practice: the social organization of clinical learning. Health & social care in the community. 2009;13:107-25.
31. Woodley L. Clinical teaching in nursing. Teaching in nursing and role of the educator. The complete guide to best practice teaching, evaluation and cirruculum development. New Yrok: Springer Publishing Company; 2013.
32. Leonardsen A, Blågestad I, Brynhildsen S, Olsen R, Gunheim-Hatland L, Gregersen A, et al. Nurses' perspectives on technical skill requirements in primary and tertiary health care. Nursin Open. 2020;00:1-7.
33. Hallin K, Danielson E. Preceptoring nursing students: registered nurses' perceptions of nursing students' preparation and study approaches in clinical education. Nurse Educ Today. 2010;30(4):296-302.
34. Brammer J. Issues in undergraduate education. RN as gatekeeper: gatekeeping as monitoring and supervision. J Clin Nurs. 2008;17:1868-76.
35. Joolaee S, Amiri S, Farahani M, Varaei S. Iranian nursing students' preparedness for clinical training: A qualitative study. Nurse Educ Today. 2015;35(10):13-7.
36. Mirzaie T, Oskouie F, Rafii F, Ravari A. Nursing students' time management: unwanted enty, marginalization of educational tasks. Iran J Nurs. 2014;26:39-50.
37. Kalyani M, Jamshidi N, Molazem Z, Torabizadeh C, Sharif F. How do nursing students experience the clinical learning environment and respond to their experiences? A qualitative study. BMJ Open. 2019;9(7):028052.
38. Donough G, Van der Heever M. Undergraduate nursing students' experience of clinical supervision. Curationis. 2018;41(1):a1833.
